# Both incompatible and compatible rhizobia inhabit the intercellular spaces of leguminous root nodules

**DOI:** 10.1080/15592324.2023.2245995

**Published:** 2023-08-13

**Authors:** Shingo Hata, Risa Tsuda, Serina Kojima, Aiko Tanaka, Hiroshi Kouchi

**Affiliations:** a Faculty of Agriculture, Ryukoku University, Otsu, Japan; b Graduate School of Bioagricultural Sciences, Nagoya University, Nagoya, Japan; c Division of Arts and Sciences, International Christian University, Mitaka, Japan

**Keywords:** apoplast, *Glycine max*, fluorescence, *Lotus japonicus*, rhizobia, root nodule

## Abstract

In addition to rhizobia, many types of co-existent bacteria are found in leguminous root nodules, but their habitats are unclear. To investigate this phenomenon, we labeled *Bradyrhizobium diazoefficiens* USDA122 and *Bradyrhizobium* sp. SSBR45 with *Discosoma* sp. red fluorescent protein (DsRed) or enhanced green fluorescent protein (eGFP). USDA122 enhances soybean growth by forming effective root nodules, but SSBR45 does not form any nodules. Using low-magnification laser scanning confocal microscopy, we found that infected cells in the central zone of soybean nodules appeared to be occupied by USDA122. Notably, high-magnification microscopy after co-inoculation of non-fluorescent USDA122 and fluorescence-labeled SSBR45 also revealed that SSBR45 inhabits the intercellular spaces of healthy nodules. More unexpectedly, co-inoculation of eGFP-labeled USDA122 and DsRed-labeled SSBR45 (and vice versa) revealed the presence of USDA122 bacteria in both the symbiosomes of infected cells and in the apoplasts of healthy nodules. We then next inspected nodules formed after a mixed inoculation of differently-labeled USDA122, without SSBR45, and confirmed the inhabitation of the both populations of USDA122 in the intercellular spaces. In contrast, infected cells were occupied by single-labeled USDA122. We also observed *Mesorhizobium loti* in the intercellular spaces of active wild-type nodules of *Lotus japonicus* using transmission electron microscopy. Compatible intercellular rhizobia have been described during nodule formation of several legume species and in some mutants, but our evidence suggests that this type of colonization may occur much more commonly in leguminous root nodules.

Leguminous plants form root nodules with compatible rhizobia, so that the rhizobia can fix atmospheric nitrogen after differentiation to bacteroids. For model legumes such as *Medicago truncatula* and *Lotus japonicus* as well as crops like soybean (*Glycine max*), it is generally known that rhizobia are delivered to plant cortical cells via root-hair infection threads and/or intercellular infection threads, and they then form symbiosomes within the infected cells.^1,2^ However, co-colonizing bacteria called “nodule endophytes”^3^ also exist, although they are much less abundant than compatible nitrogen-fixing rhizobia. More than ten and thirty genera of nodule-inducing and non-rhizobial endophytes, respectively, have been described in the root nodules of various legumes.^4–6^ Of these, some bacteria have been reported to promote plant growth,^7^ to produce anti-microbial compounds,^8^ to act as plant pathogens,^9,10^ to suppress plant pathogens,^11^ and to suppress so-called “cheater” rhizobia.^12^ However, most bacteria do not exhibit detectable functions. Nodules are thought to be frequent infection sites with weak defense reactions, which create diffusion barriers that prevent spreading of endophytes throughout whole plants.^9^ In this paper, we report an investigation of the habitats of these bacteria. First, we examined whether or not *Bradyrhizobium* sp. SSBR45 (BioSample: SAMD00561014 and SAMD00561015), isolated from *Aeschynomene indica*,^13^ is a nodule endophyte of soybean. During the course of experiments, we noticed that both the incompatible SSBR45 but also the compatible nitrogen-fixing bacterium *Bradyrhizobium diazoefficiens* (*i.e*., reclassified from *Bradyrhizobium japonicum*) USDA122 (BioSample: SAMD00022949)^14^ were present in the intercellular spaces of soybean nodules. We also confirmed that compatible *Mesorhizobium loti* Tono (BioSample: SAMD00055564) colonizes the apoplasts of wild-type *Lotus japonicus* nodules.

We conducted inoculation experiments in which we transplanted a single sterile soybean seedling (*G. max* L. cv. Fukuyutaka) into each of the sterilized Leonard jar assembly containing vermiculite and nitrogen-free medium.^13^ Next, 2 × 10^6^ cells/plant of *B. diazoefficiens* USDA122 and/or 2 × 10^6^ cells/plant of *Bradyrhizobium* sp. SSBR45 were inoculated immediately following transplantation, and the plants were then grown in a greenhouse as described previously.^13^ Fluorescence-labeling of USDA122 and SSBR45 was also performed as previously described.^13^ The soybean nodules were harvested at around 4 weeks post-inoculation. For laser scanning confocal microscopy, the nodules were embedded in 5% agar, from which we prepared semi-thin sections of 80 µm thickness.^13^ These were visualized using a TCS SP8 DMi8 microscope (Leica, Heidelberg, Germany) equipped with a HCX PL APO CS2 10×/0.40 objective lens (Leica) and a HCX PL APO 63×/1.30 GLYC oil immersion objective lens (Leica) for low- and high-powered magnification, respectively. *Discosoma* sp. red fluorescent protein (DsRed) and enhanced green fluorescent protein (eGFP) were excited and detected as previously reported.^13^ The captured images were processed using Photoshop software (Adobe Systems, San Jose, CA). *L. japonicus* B129 Gifu and MG-20 Miyakojima were also grown with *M. loti* Tono under monoxenic conditions as described above for soybean, except that a *L. japonicus* seedling was transplanted into a disposable vinyl pot containing vermiculite and nitrogen-free medium. Transmission electron microscopy was also performed as described previously.^15^

We were originally interested in endophytic nature of *Aeschynomene* symbionts in rice roots.^16^ However, the wide recognition of nodule endophytes as described above prompted us to examine whether incompatible SSBR45 is a nodule endophyte of soybean. For an initial experiment, we checked that the “autofluorescence” of root nodules formed by non-fluorescent USDA122 did not affect the detection of DsRed or eGFP under the conditions we used for laser scanning confocal microscopy (see: insets of Supplementary Figure S1c and f). Next, at low magnification we observed the interior of soybean nodules formed by fluorescence-labeled USDA122, the *nodABC* genes of which,^14^ essential for Nod factor synthesis, are 100% identical at the nucleotide level to those of well-studied USDA110 (BioSample: SAMD00008651).^17,18^ As expected, the central tissue consisted of both infected and non-infected cells. The former were filled with DsRed- or eGFP-labeled USDA122 (Supplementary Figure S1). We also inoculated SSBR45 by itself on soybean roots, and this resulted in no nodule formation. Although it was reported that *Bradyrhizobium* sp. ORS278 (Biosample: PRJNA19575)^19^—which was originally isolated from African *Aeschynomene sensitiva* and had an average nucleotide identity of 87% to SSBR45^13^—formed bacteria-free bumps,^20^ we did not observe this morphological change in soybean roots in this study. The reason for this discrepancy is unclear, but the bumps themselves were thought to be caused by phytohormones.^20^ Thus, the quality and/or quantity of phytohormones synthesized by the two strains may be different.

As a preliminary experiment of co-inoculation, we confirmed that USDA122 and SSBR45 did not exert any antagonistic effect on each other’s growth in HEPES-MES （HM） liquid medium^21^ *in vitro* (Supplementary Figure S2). However, whether they show competitive infection to plant roots remains to be clarified in the future. We co-inoculated non-fluorescent USDA122 and fluorescence-labeled SSBR45 onto soybean roots. Various fluorescence intensities were observed in the resulting nodules. Five out of 17 nodules fluoresced strongly and were obviously different from the non-fluorescent control, such as those shown in Figure 1a. In contrast, the other 12 nodules fluoresced weakly and appeared more similar to the negative control (insets of Supplementary Figure S1c and f), such as those shown in Figure 1d. In the former case, we note that fluorescence was detected not only in central infected tissues but also in peripheral cortical tissues. Conclusively, images taken under high-powered magnification indicated that fluorescence-labeled SSBR45 inhabited the intercellular spaces of soybean nodules (Figures 1b, c, e & 1f). Moreover, the co-inoculation of DsRed- and eGFP-labeled SSBR45 together with non-fluorescent USDA122 strongly confirmed the above results (Figure 1g–i). Thus, SSBR45 is an endophyte of soybean nodules.

**Figure 1.**
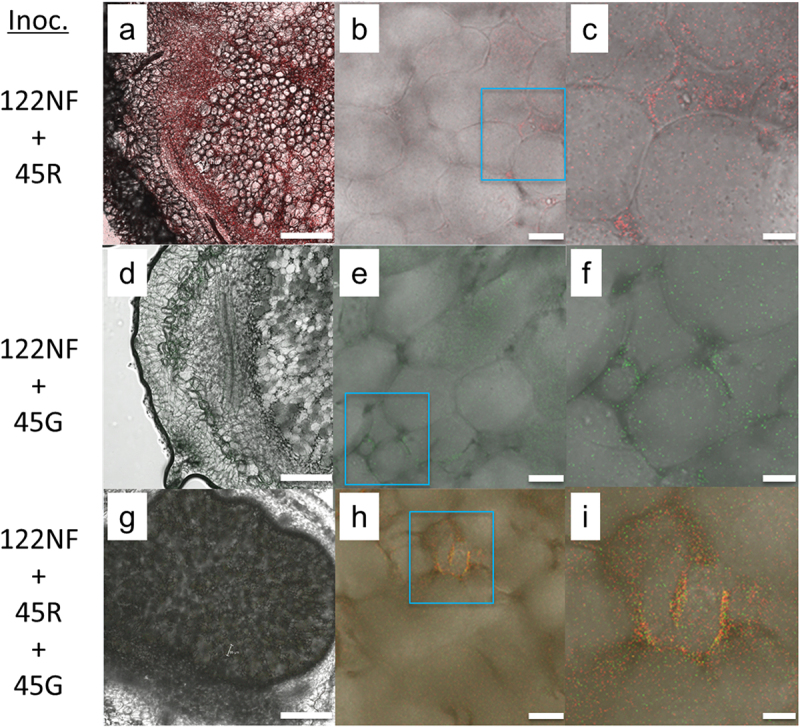
*Bradyrhizobium* sp. SSBR45 observed in the apoplasts of soybean root nodules. Nodules were formed following co-inoculation of equal numbers of non-fluorescent *B. diazoefficiens* USDA122 and DsRed-labeled *Bradyrhizobium* sp. SSBR45 (a-c; 122NF +45 R; overlay of DsRed and bright-field images), those inoculated by non-fluorescent USDA122 and eGFP-labeled SSBR45 (d-f; 122NF +45 G; overlay of eGFP and bright-field images), and those inoculated by all three strains (g-i; 122NF +45 R +45 G; overlay of DsRed, eGFP, and bright-field images). Panels (c), (f), and (i) are zoomed-in images of the square zones indicated in (b), (e), and (h), respectively. The yellow color shown in panels (g-i) indicates the mixture of red and green fluorescence. Scale bars: (a), (d), and (g), 250 µm; (b), (e), and (h), 25 µm; and (c), (f), and (i), 10 µm.

We also examined the location of SSBR45 using differently-labeled USDA122 and SSBR45. As expected, when mature nodules were formed following co-inoculation of eGFP-labeled USDA122 and DsRed-labeled SSBR45, SSBR45 bacteria were found in the intercellular spaces of cortical cells (Supplementary Figure S3a-c). However, during the course of this observation, we also noticed that both incompatible SSBR45 bacteria and compatible USDA122 bacteria were present in intercellular spaces (Supplementary Figure S3c). To confirm the presence of compatible USDA122 in the apoplasts, we then observed the insides of nodules formed by an equal mixture of DsRed-labeled USDA122 and eGFP-labeled USDA122, together in the absence of SSBR45. It is well-known that, following co-inoculation of two differently-labeled rhizobia, most infection threads are occupied by a single rhizobium^22,23^ and also that most of the resulting nodules appear as though they consisted of clonal rhizobium.^13,22,24–26^ In our case, 12 and 10 nodules were observed, in which only DsRed-labeled USDA122 and eGFP-labeled USDA122, respectively, occupied their infected cortical cells. Figure 2 shows the inside of a mature nodule, the infected cells of which were occupied only by DsRed-labeled USDA122. We observed the overlay of DsRed, eGFP, and bright-field images. It is noteworthy that both DsRed-labeled USDA122 and eGFP-labeled USDA122 were scattered throughout the intercellular spaces of this nodule (Figure 2b). It is also worth noting that fluorescence in the apoplasts was much weaker than in symbiosomes. GroEL4, in combination with GroEL3, was reported to contribute most of the GroEL chaperonin pool found in *Bradyrhizobium* bacteroids.^27^ Therefore, the *BjGroEL4* promoter should be strong in bacteroids and could be suitable for their observation. Regrettably, this promoter is not suitable for detection of bacteria in apoplasts. Nevertheless, we were able to identify the inhabitation of compatible USDA122 bacteria in intercellular spaces. We also examined an emerging young nodule formed by DsRed-labeled USDA122 and eGFP-labeled SSBR45. In this emerging immature nodule, USDA122 bacteria were again detected in the apoplasts (Supplementary Figure S3f and g). SSBR45 bacteria were also detected, but their eGFP signal was very low compared to the DsRed signal of USDA122. Overall, these results indicate that intercellular colonization of USDA122 and SSBR45 occurs rather early in nodule formation.

**Figure 2.**
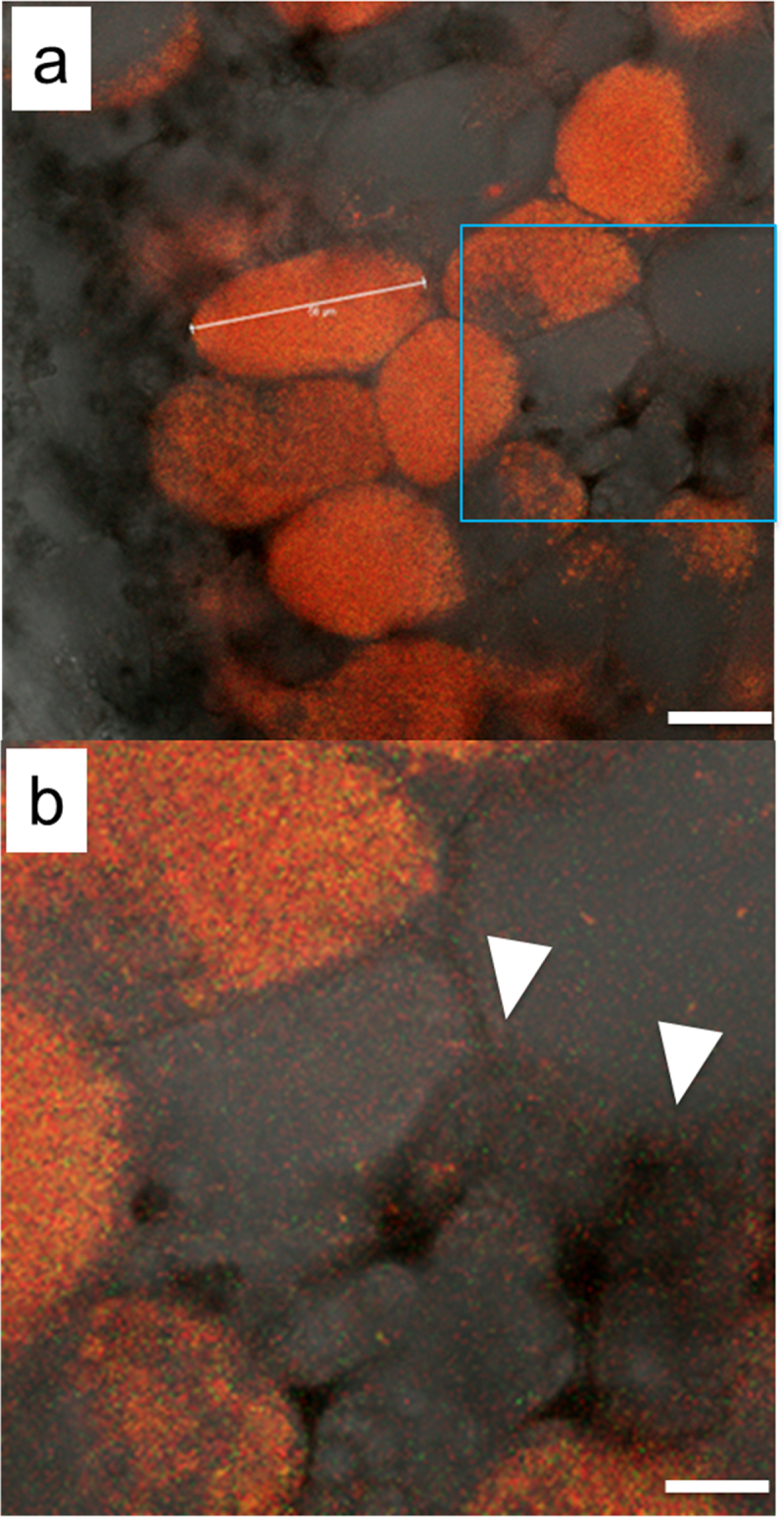
Overlay of DsRed, eGFP, and bright-field images of a root nodule formed by the inoculation of almost equal numbers of DsRed-labeled USDA122 and eGFP-labeled USDA122, in the absence of SSBR45. Panel (b) is a zoomed-in image of the square zone indicated in (a). In this nodule, infected cells were occupied only by DsRed-labeled USDA122. Arrowheads indicate intercellular spaces where USDA122 bacteria were observed. Scale bars: (a), 25 µm, and (b), 10 µm.

Giraud *et al*. found that the Nod factor-producing *Bradyrhizobium* sp. ORS285 (BioSample: SAMEA103957792), originally isolated from *Aeschynomene*,^19^ colonizes the intercellular spaces of soybean nodules “in rare cases,” as well as the symbiosomes of infected cortical cells.^20^ Similarly, Okazaki *et al*. reported that *Bradyrhizobium elkanii* USDA61 (BioSample: SAMD00024454) appeared to have infected soybean roots intercellularly and then formed nodules. In this case, nodule formation occurred in a type III secretion system-dependent and Nod factor-independent manner, without root-hair curling or infection thread formation.^28^ To our knowledge, these are the only two reports of apoplastic colonization of compatible rhizobia in soybean nodules. The results of this study substantiate that the intercellular inhabitation of compatible rhizobia occurs.

Using spontaneous nodule mutants of *L. japonicus* elegantly, Madsen *et al*. proposed that Nod factor-independent intercellular infection, crack entry, and Nod factor-dependent infection thread formation occurred in legume evolution in this order. They also postulated that these invasion modes were maintained during evolution and are not mutually exclusive.^29^ However, whether or not typical intercellular infection occurs between wild-type *M. loti* bacteria and wild-type *L. japonicus* has not been described. Therefore, for a final experiment, we examined the presence or absence of *M. loti* Tono in the apoplasts of wild-type *L. japonicus* nodules using transmission electron microscopy. As shown in Figure 3a, b. *M. loti* bacteria were found in the intercellular spaces of *L. japonicus* nodules. It is noteworthy that the most bacteria present in apoplasts were not enveloped by peribacteroid membranes, in contrast to those found in symbiosomes. Nevertheless, a bacterium indicated by a yellow arrow in Figure 3b may be surrounded by an incomplete membrane-like structure. At present, we have no knowledge about the bacterium. Some bacteria were surrounded by an electron-dense matrix material (Figure 3c). This dense material is thought to be composed of glycoproteins and is important for the intercellular uptake of bacteria into plant cortical cells.^30^ The “peg”-like structures containing this material have been described during nodule formation in *Mimosa*,^31^ white lupine,^32,33^ *Phaseolus*,^34^ *L. japonicus* mutants,^29^ and *Lotus bruttii*.^35^ Thus, as predicted by Madsen *et al*.,^29^ it is probable that the intercellular infection of wild-type *M. loti* occurs in wild-type *L. japonicus*, although its efficiency is much lower than via the infection thread pathway.

**Figure 3.**
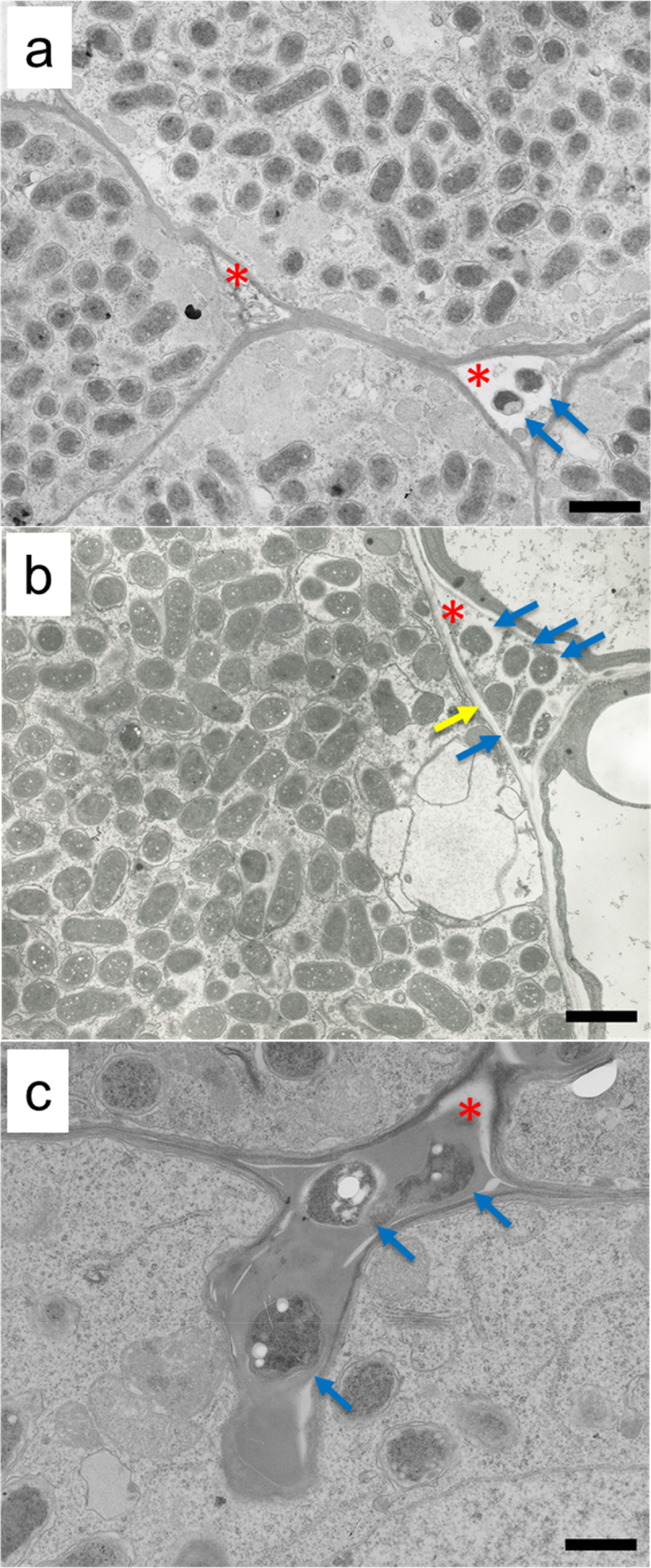
Mesorhizobium loti Tono in the root nodules of *Lotus japonicus* as observed by transmission electron microscopy. (a), A nodule of *L.* *japonicus* B129 Gifu at 8 days post-inoculation; and (b) *L.* *japonicus* MG-20 Miyakojima at 14 days post-inoculation.　(c) Intercellular bacteria surrounded by electron-dense compounds, the main components of which are thought to be hydroxyproline-rich glycoproteins. These were found in a nodule of *L.* *japonicus* B129 Gifu at 8 days post-inoculation. Red asterisks indicate intercellular spaces; blue arrows indicate bacteria without surrounding membranes; yellow arrow indicates a bacterium surrounded with an incomplete membrane-like structure. Scale bars: (a) and (b), 2 µm; and (c), 1 µm.

Using soybean, we showed that both incompatible SSBR45 and compatible USDA122 inhabit the intercellular spaces of root nodules. The apoplastic colonization of compatible rhizobia seems to have been overlooked thus far but may be widespread or even common in leguminous root nodules, as is the colonization of nodule endophytes. The results of this work also support the notion that *M. loti* performs intercellular colonization of the nodules of *Lotus* species. The bacterial intercellular invasion of plant roots has been described in several legumes and in non-leguminous species (*e.g.*, actinorhizal plants and *Parasponia*, as reviewed by Ibáñez *et al*.).^36^ Sprent and James estimated that about 25% of legume species undergo infection thread-independent colonization of rhizobia but, interestingly, they categorized both *Glycine* and *Lotus* as infection thread-dependent genera.^37^ However, as a matter of course, the intercellular inhabitation of a rhizobium does not necessarily indicate that it possesses the ability to perform intercellular infection of plant cells. Whether or not compatible rhizobia, such as USDA122, perform this type of intercellular infection in soybean roots remains to be elucidated by future research.

How did the observed bacteria reach the intercellular spaces? Since a number of epidermal cracks have been reported to form on the surface of nodule primordia,^35^ both the endophyte SSBR45 and compatible USDA122 may have entered soybean nodules directly following attraction to sugars, amino acids, and mucilage, etc. secreted from these cracks. However, in the case of *L. japonicus*, an endophyte *Rhizobium mesosinicum* strain KAW12 has also observed to be guided within infection threads initiated by compatible *M. loti*.^38^ Moreover, the pathogenic endophyte *Ralstonia solanacearum* strain GMI1000 entered *M. truncatula* nodules from root tips through the vasculature.^9^ Therefore, future research is required to determine the route of infection. In any case, successive checkpoints exist during intercellular infection,^35^ and that is why some nodule endophytes can colonize intracellularly^12,38^ while others – like SSBR45—cannot.

Some rhizobia are also known to undergo terminal bacteroid differentiation (TBD), especially in the nodules of the so-called inverted repeat-lacking clade (IRLC) of legumes.^39^ TBD confers benefits to the host plants,^40^ but reduces the proliferative capacity of rhizobia greatly. In such cases, intercellular colonization would be beneficial for the rhizobial prosperity of descendants. Moreover, even in the nodules of non-IRLC legumes that do not undergo obvious TBD, intercellular colonization may be beneficial for the survival of rhizobia in natural environments.

## Supplementary Material

Supplemental Material
