# Supplementary figures and images for: Both incompatible and compatible rhizobia inhabit the intercellular spaces of leguminous root nodules
Source: Plant Signal Behav. 2023 Aug 13;18(1):2245995. doi: 10.1080/15592324.2023.2245995 (PMC10424618; doi:10.1080/15592324.2023.2245995)

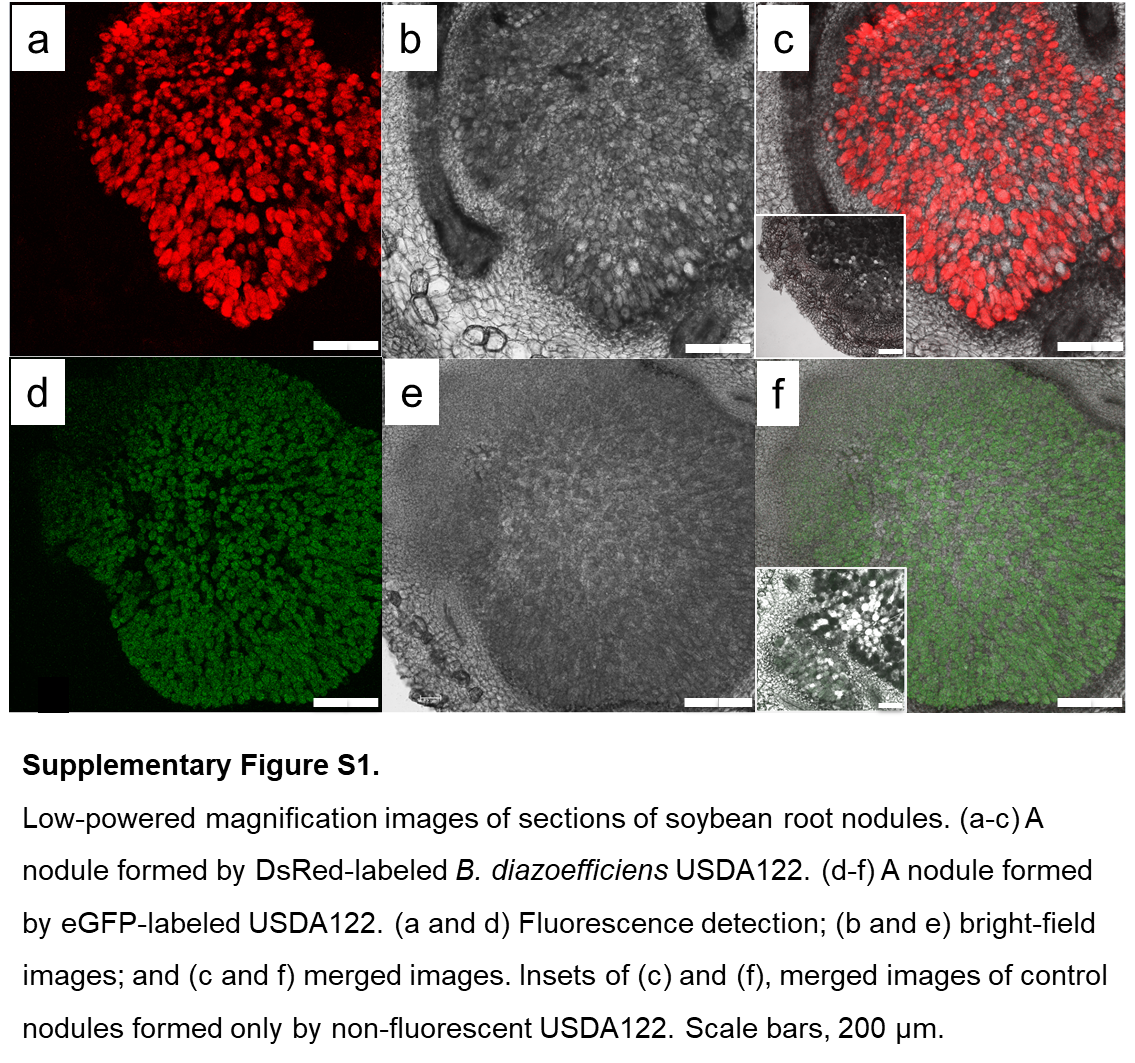

Supplement: Supplemental Material [file KPSB_A_2245995_SM9476.zip › HATA2_SupplFig S1.docx]

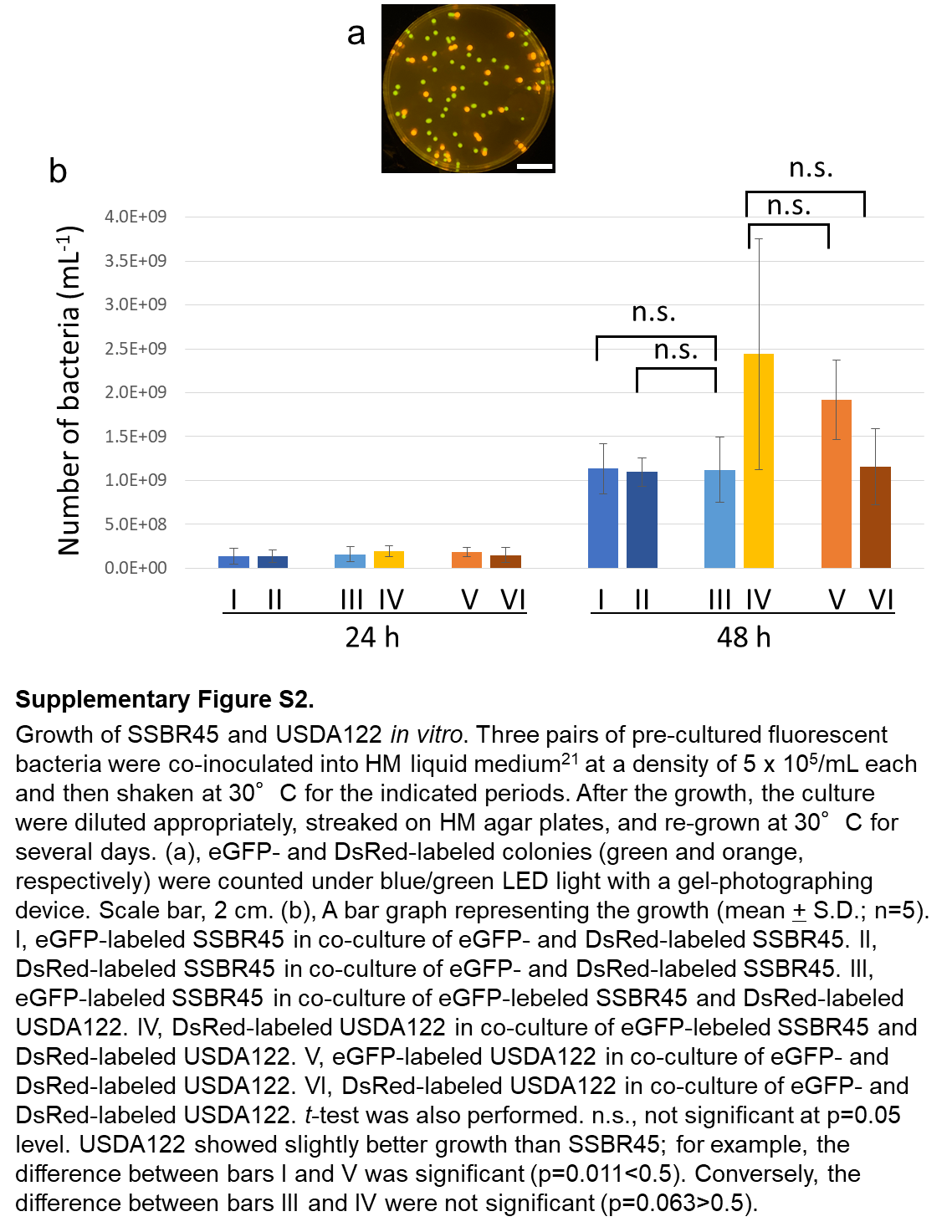

Supplement: Supplemental Material [file KPSB_A_2245995_SM9476.zip › HATA2_SupplFig S2.docx]

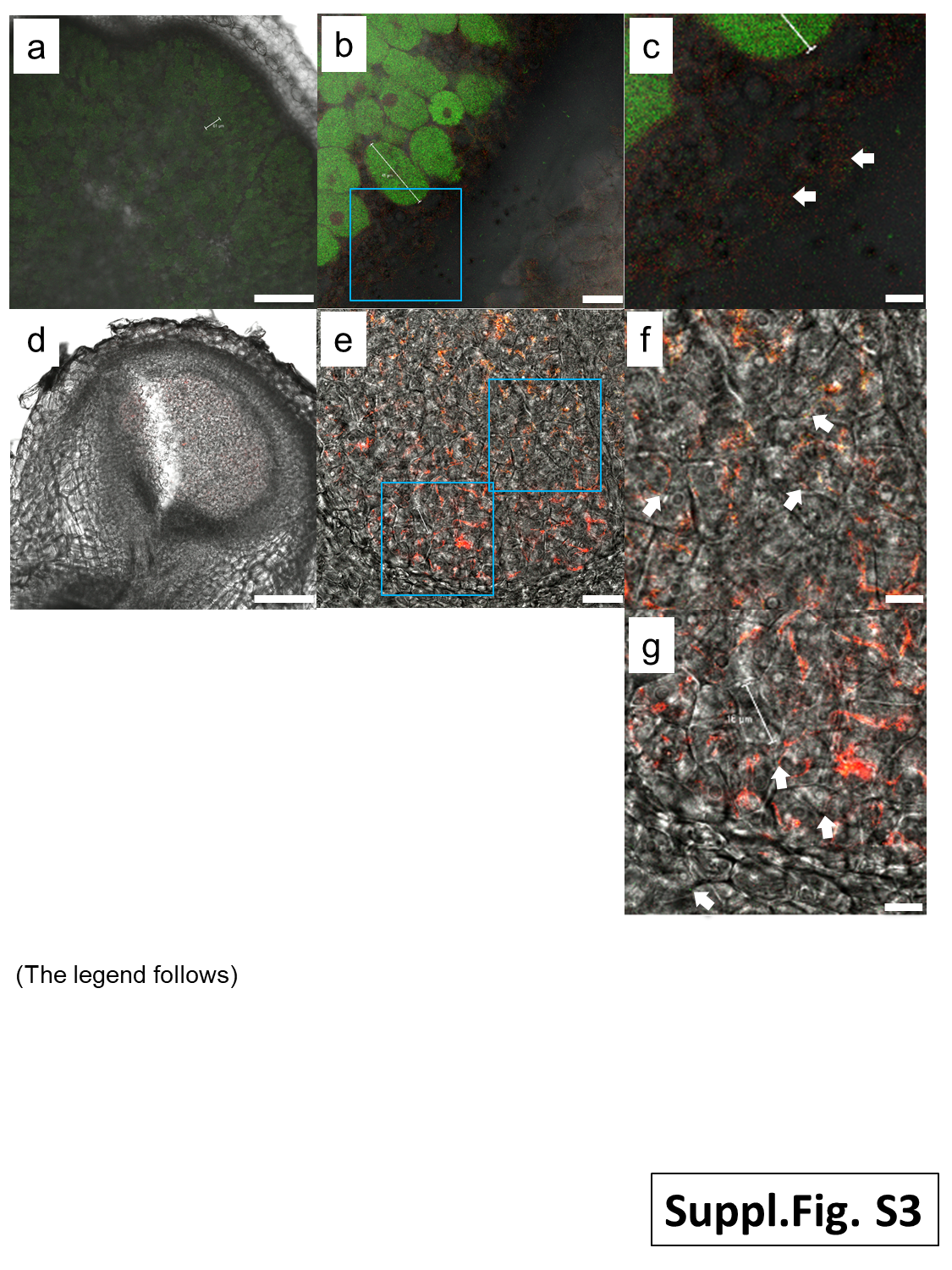

Supplement: Supplemental Material [file KPSB_A_2245995_SM9476.zip › HATA2_SupplFig S3.docx]

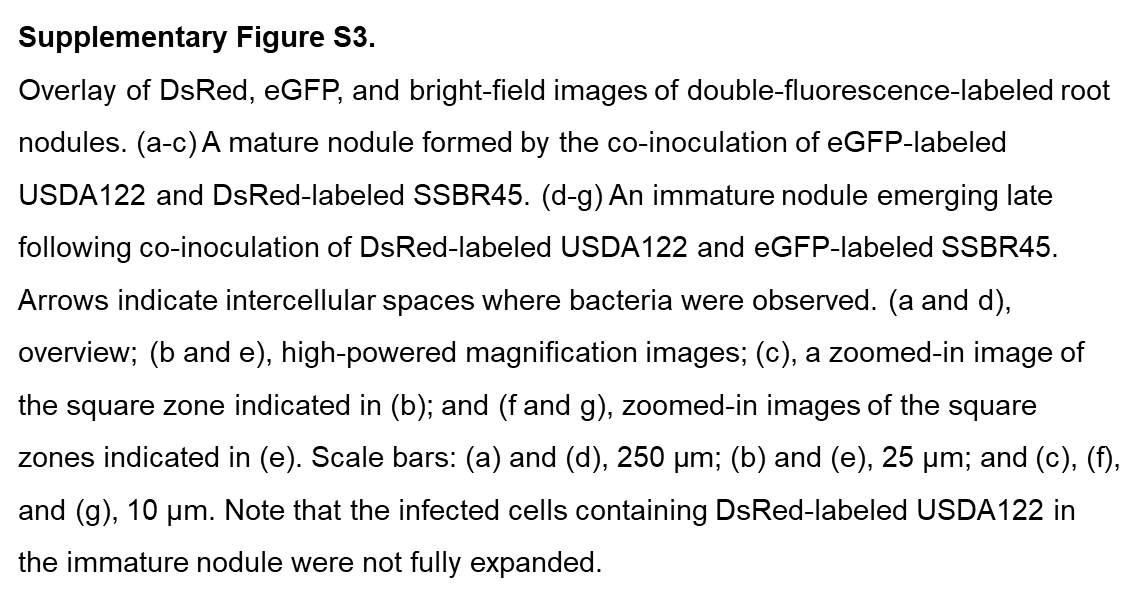

Supplement: Supplemental Material [file KPSB_A_2245995_SM9476.zip › HATA2_SupplFig S3Legend.docx]
